# Do peripheral and/or central chemoreflexes influence skin blood flow in humans?

**DOI:** 10.14814/phy2.12181

**Published:** 2014-10-24

**Authors:** Matthew J. Heffernan, Matthew D. Muller

**Affiliations:** 1 Pennsylvania State University College of Medicine, Penn State Hershey Heart and Vascular Institute, Hershey, Pennsylvania

**Keywords:** Blood pressure, chemoreceptors, cutaneous vasoconstriction, heart rate, muscle blood flow, sympathetic activation

## Abstract

Voluntary apnea activates the central and peripheral chemoreceptors, leading to a rise in sympathetic nerve activity and limb vasoconstriction (i.e., brachial blood flow velocity and forearm cutaneous vascular conductance decrease to a similar extent). Whether peripheral and/or central chemoreceptors contribute to the cutaneous vasoconstrictor response remains unknown. We performed three separate experiments in healthy young men to test the following three hypotheses. First, inhibition of peripheral chemoreceptors with brief hyperoxia inhalation (100% O_2_) would attenuate the cutaneous vasoconstrictor response to voluntary apnea. Second, activation of the peripheral chemoreceptors with 5 min of hypoxia (10% O_2_, 90% N_2_) would augment the cutaneous vasoconstrictor response to voluntary apnea. Third, activation of the central chemoreceptors with 5 min of hypercapnia (7% CO2, 30% O_2_, 63% N_2_) would have no influence on cutaneous responses to voluntary apnea. Studies were performed in the supine posture with skin temperature maintained at thermoneutral levels. Beat‐by‐beat blood pressure, heart rate, brachial blood flow velocity, and cutaneous vascular conductance were measured and changes from baseline were compared between treatments. Relative to room air, hyperoxia attenuated the vasoconstrictor response to voluntary apnea in both muscle (−16 ± 10 vs. −40 ± 12%, *P* = 0.023) and skin (−14 ± 6 vs. −24 ± 5%, *P* = 0.033). Neither hypoxia nor hypercapnia had significant effects on cutaneous responses to apnea. These data indicate that skin blood flow is controlled by the peripheral chemoreceptors but not the central chemoreceptors.

## Abstract (precis)

Voluntary apnea activates the central and peripheral chemoreceptors, leading to a rise in sympathetic nerve activity and limb vasoconstriction. We performed three separate experiments in healthy young men to determine if central and/or peripheral chemoreceptors contribute to this vasoconstrictor response. The data indicate that skin blood flow is controlled by the peripheral chemoreceptors but not the central chemoreceptors.

## Introduction

Stimulation of the central and peripheral chemoreceptors by low arterial oxygen concentration and/or high carbon dioxide initiates changes in ventilation accompanied by reflex increases in muscle sympathetic nerve activity, heart rate (HR), and blood pressure (BP; Hardy et al. 1994; Leuenberger et al. 2001; Patel et al. 2013). Limb vascular responses to chemoreflex activation are a balance between sympathetic vasoconstriction and metabolic vasodilation. Skeletal muscle vasodilates in response to continuous hypoxia and hypercapnia (Kontos et al. 1967; Leuenberger et al. 1999; Pollock et al. 2014), but vasoconstricts during a voluntary apnea (Katragadda et al. 1997; Imadojemu et al. 2002; Clanton 2007). Prior studies have shown that chemoreceptor activation elicits altered cardiovascular responses in patients with heart failure, obstructive sleep apnea, obesity, and hypertension (Narkiewicz et al. 1999a,b,c). Thus, understanding how chemoreflexes influence the human circulation has potential clinical relevance.

One prevailing concept is that the cutaneous circulation is a general model of the entire cardiovascular system (Holowatz et al. 2008) and can be used to understand pathophysiology. However, this opinion is not universally accepted (Cui 2008; Stewart 2008) and it should be noted that skin blood flow and muscle blood flow serve different physiologic purposes and are differentially controlled by the sympathetic nervous system (Delius et al. 1972a,b; Bini et al. 1980). With respect to sympathetic vasoconstriction, prior studies have shown that baroreflex activation via lower body negative pressure elicits a reduction in both muscle blood flow and skin blood flow (Beiser et al. 1970; Johnson et al. 1976; Tripathi and Nadel 1986; Crandall et al. 1996; Brothers et al. 2010). Moreover, Patel et al. (2014) showed that chemoreflex activation with voluntary apnea caused similar reductions in both skin blood flow and muscle blood flow. These studies listed above suggest that sympathetic activation causes similar changes in the skin and skeletal muscle circulations. However, we recently found that age‐associated differences in skeletal muscle hypoxic vasodilation were not observed in the skin (Pollock et al. 2014), thus demonstrating a situation in which skin and muscle do not respond the same. Currently, it remains unclear if peripheral and/or central chemoreceptors participate in reflex control of the cutaneous circulation.

**Figure 1.**
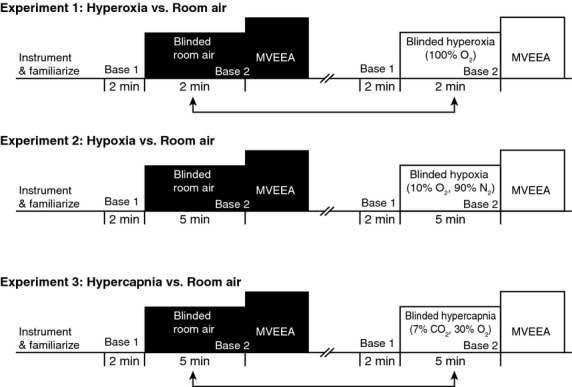
Procedure overview for Experiments 1, 2, and 3. The arrows indicate that trials were randomized. Please see text for details. Base, baseline; MVEEA, maximal voluntary end expiratory apnea.

The purpose of this study was to evaluate the forearm vascular responses (skin vs. muscle) in response to peripheral and central chemoreflex activation. In three separate studies, we evaluated forearm vasoconstrictor responses to voluntary apnea under conditions of altered chemoreceptor input. In Experiment 1, we tested the hypothesis that brief hyperoxia (100% O_2_) would attenuate the cutaneous vasoconstrictor response to voluntary apnea compared to room air (21% O_2_, 79% N_2_). In Experiment 2, we tested the hypothesis that hypoxia (10% O_2_, 90% N_2_) would augment the cutaneous vasoconstrictor response to voluntary apnea. In Experiment 3, we tested the hypothesis that hypercapnia (7% CO_2_, 30% O_2_, 63% N_2_) would have no influence on the cutaneous responses to voluntary apnea (Fig. 1).

## Materials and Methods

### Experimental design and subjects

In this experiment, maximal voluntary end expiratory apneas (MVEEA) were performed during the conditions of room air breathing (21% oxygen), hyperoxia (100% O_2_), hypoxia (10% O_2_), and hypercapnia (7% CO_2_%, 30% O_2_) to determine whether forearm cutaneous vascular responses are under central and/or peripheral chemoreflex control. Hyperoxia is known to inhibit the peripheral chemoreceptors (also called “carotid chemoreceptors” or “arterial chemoreceptors”; Lahiri et al. 1981; Stickland et al. 2008), whereas hypoxia augments peripheral chemoreceptors (Lahiri and DeLaney 1975). Hypercapnia stimulates central chemoreceptors (Lahiri et al. 1981; Somers et al. 1989). Because lung inflation inhibits sympathetic outflow, voluntary apneas are commonly used in research studies to isolate the underlying reflex effects on the vasculature. It should also be noted that voluntary apneas cause a reduction in arterial oxygen saturation (SaO_2_) and a rise in CO_2_ (Elsner et al. 1971; Morgan et al. 1993; Khayat et al. 2004; Steinback et al. 2010; Seitz et al. 2013). We recently demonstrated moderate to high test–retest reliability with regard to physiological responses to MVEEA (Patel et al. 2014).

All study protocols were approved in advance by the Institutional Review Board of the Penn State Milton S. Hershey Medical Center and conformed to the Declaration of Helsinki. A total of 14 young healthy men volunteered to participate and provided written informed consent. Demographic information can be found in Table 1. Women were excluded due to established sex differences in vascular control during hypoxic stress (Casey et al. 2014; Patel et al. 2014). Six male subjects participated in Experiment 1, nine subjects participated in Experiment 2, and seven subjects participated in Experiment 3. Two subjects performed all three experiments, one subject performed both Experiments 1 and 2, and three subjects performed both Experiments 2 and 3. Control trials were always repeated in these individuals. The sample size for Experiment 1 was calculated once the first four subjects had completed testing. Specifically, we determined that if the mean difference in cutaneous vascular conductance (CVC) between room air and hyperoxia in response to apnea was 10% with a standard deviation of 6%, we would need to enroll five subjects to be able to reject the null hypothesis with a probability of 0.80 and a type 1 error of 0.05. All subjects had supine resting blood pressures below 120/80 mmHg and were nonasthmatic, nonobese, nonsmokers, not taking any prescription, or vasoactive medication, not routinely exposed to carbon monoxide, and were in good health as determined by history and physical examination. All were physically active but none were competitive athletes. Subjects refrained from caffeine, alcohol, and exercise for 24 h before the study and arrived to the laboratory in a semifasted state (i.e., 4–6 h after their last meal).

**Table 1.** Demographic information.

	Experiment 1	Experiment 2	Experiment 3
Sample size	6	9	7
Age (years)	27 ± 1	28 ± 1	28 ± 1
Height (cm)	180 ± 2	179 ± 2	184 ± 2
Weight (kg)	82.3 ± 3	83.7 ± 4	87.7 ± 2
BMI (kg/m^2^)	25.4 ± 1	26.0 ± 1	25.8 ± 1
MAP (mmHg)	80 ± 1	76 ± 2	78 ± 2
HR (bpm)	57 ± 4	56 ± 3	54 ± 4

BMI, body mass index; MAP, mean arterial pressure; HR, heart rate.

### Physiological measurements

All experiments were conducted in a dimly lit, thermoneutral laboratory (22–25°C). Subjects were supine and clothed in a high‐density tube‐lined suit (Med‐Eng Systems, Ottawa, ON, Canada) that covered the entire body except for the feet, hands, both forearms, and head. Neutral water (34–35°C) was perfused through the suit to maintain mean skin temperature at a constant level and ensure the obtained responses were not confounded by changes in ambient temperature. Upon arrival to the laboratory, subjects were outfitted with a 3‐lead EKG (Cardiocap/5; GE Healthcare, Waukesha, WI) to monitor HR and a finger blood pressure cuff (Finometer, FMS). Prior to the protocol, resting blood pressures were obtained in triplicate by automated oscillometry of the left brachial artery (Philips Sure Signs VS3, Andover, MA) after 15 min of quiet rest. These values were used to verify the Finometer pressures.

We used Doppler ultrasound (GE Vivid 7) to record brachial blood flow velocity in the left arm as previously described (Wilson et al. 2007). Briefly, a linear transducer was placed over the brachial artery and the insonation angle was <60°. Brachial artery mean blood flow velocity (BBFV) was acquired in pulsed Doppler mode and velocity waveforms were synchronized to a data acquisition system (PowerLab; ADInstrument, New Castle, Australia) by a Doppler audio transformer (Herr et al. 2010). Arterial diameter measurements were not measured in this study because our equipment does not allow for the simultaneous measurement of velocity and diameter. We recently showed that brachial artery diameter does not change in response to MVEEA (Patel et al. 2014). Cutaneous blood flow flux was measured by three laser Doppler probes placed on the right ventral forearm. The local temperature of the forearm skin measurement sites was maintained at 34°C by a local heater which also held the laser in place (Moor Instruments, Wilmington, DE).

Subjects were outfitted to measure respiratory parameters. A tight sealing oronasal facemask was fitted on the subject before trials began. Arterial blood oxygen concentration (SaO_2_) was measured with an ear clip (RGM 5250; Ohmeda, Madison, WI). The RGM was also used to measure end tidal carbon dioxide (ETCO2), and minute ventilation. Inspiration and expiration were continuously monitored with a pneumobelt which also allowed for offline analysis of apnea onset and offset.

### Experiment 1: Voluntary end expiratory apneas under hyperoxia versus room air

All subjects underwent familiarization trials to ensure that they performed end expiratory apneas (holding their breath with the lungs empty to avoid physiologic activity associated with inflation of the lungs). Baseline measurements of HR, BP, SaO_2_, minute ventilation, ETCO2, CVC, and BBFV were monitored for 2 min. Following the baseline, 2 min of hyperoxia (100% O_2_) or room air was administered to the facemask from a large reservoir bag. Hyperoxia was administered to temporarily inhibit the peripheral chemoreceptors (Lahiri et al. 1981; Stickland et al. 2008). At the end of exposure to either hyperoxia or room air, the subjects were instructed to remain relaxed, and perform the MVEEA. After a recovery period of 5–10 min, the opposite gas was used for the next trial. Gases were administered in a counterbalanced, single‐ blinded fashion. However, all subjects were able to detect when they received 100% oxygen since the apnea duration was longer.

### Experiment 2: Voluntary end expiratory apneas under hypoxia versus room air

The same measurements as Experiment 1 were obtained, but room air was used for the first trial followed by hypoxia (10% O_2_, 90% N_2_). Hypoxia was administered to activate peripheral chemoreceptors (Lahiri et al. 1981; Stickland et al. 2008). The order was not counterbalanced based on the long‐term hemodynamic changes associated with breathing hypoxic gas (Xie et al. 2001). The subjects were not told ahead of time which gas would be given but they were able to detect the hypoxia because of the increased ventilation they experienced.

### Experiment 3: Voluntary end expiratory apneas under hypercapnia versus room air

The same measurements as Experiment 1 and 2 were obtained, but the subjects breathed either 5 min of hypercapnia (7% CO_2_, 30% O_2_, 63% N_2_) or room air prior to the MVEEA. Hypercapnia was administered to activate central chemoreceptors (Lahiri et al. 1981; Somers et al. 1989). Consistent with previous hypercapnia studies (Simmons et al. 2007), slight hyperoxia was used to prevent activation of peripheral chemoreceptors (Izdebska et al. 1996). The gases were administered in a counterbalanced, single‐blinded fashion. The subjects were not told ahead of time which gas would be given but they were able to detect the hypercapnia because of the increased ventilation they experienced.

#### Data collection and statistical analysis

All data were collected using PowerLab (ADInstruments) that was set at 200 Hz recording speed, and analyzed offline. Using PowerLab, we quantified duration of the MVEEA, lowest O_2_ saturation, HR, mean arterial pressure (MAP), CVC, and BBFV. The skin blood flow flux from the three forearm skin blood flow lasers was averaged and presented as CVC, calculated as flux/MAP and expressed as a percent change from Base 2. BBFV was also calculated as a percent change in order to compare CVC and BBFV in the same units, consistent with our recent publication (Patel et al. 2014). Forearm vascular conductance index (FVCI) was calculated as BBFV/MAP. We analyzed six different time points, detailed below.

*Base 1:* 20‐sec period of stable room air baseline (before administration of gas)
*Base 2:* The last 20 sec of the chosen gas before the apnea occurred.
*Apnea 1 (A1):* The first three cardiac cycles (detected from the Finometer waveform) of the apnea after the subject had completely emptied their lungs (absence of lung inflation but no reduction in SaO_2_ relative to Base 2).
*Apnea 2 (A2):* The last three cardiac cycles before the subject inhaled (called the “asphyxic break point) at the end of the apnea (absence of lung inflation and a reduction in SaO_2_ relative to Base 2).
*Recovery 1 (R1):* The first three cardiac cycles after the subject resumed breathing.
*Time Matched Control:* Because the gases affected the duration of the apnea, a three cardiac cycle “time matched control” was also analyzed to ensure that the same physiologic conditions were being compared. For example, if a person had a breath hold duration of 50 sec with hyperoxia and 30 sec with room air, the hyperoxia “time matched control” would include a bin of three cardiac cycles from about 27–30 sec and this would be compared to the room air A2 (i.e., the last three cardiac cycles prior to inspiration).

**Figure 2.**
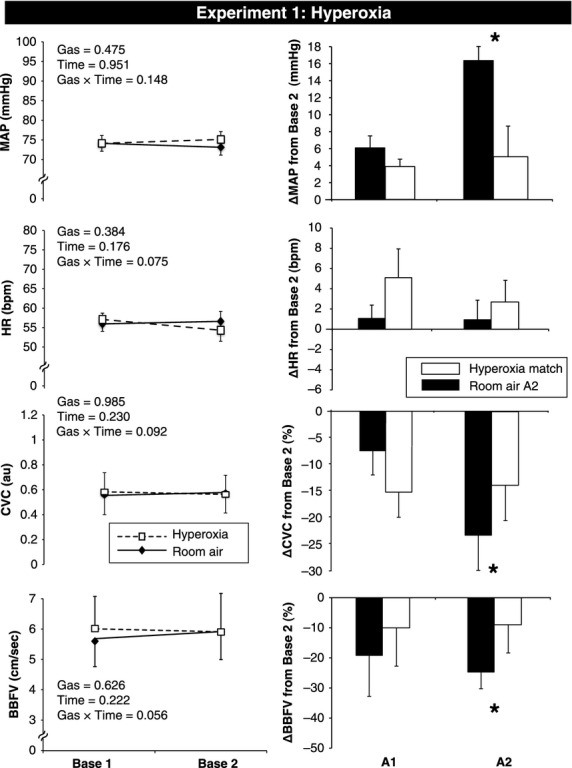
Hemodynamic and vascular changes in response to hyperoxic gas and room air apnea in Experiment 1. Absolute changes from Base 1 (room air) to Base 2 (at the end of hyperoxia or room air administration) are presented in the left panel. Room air is represented by a solid line, and hyperoxia is represented by a dashed line. Changes from Base 2 are presented in the right panel for both A1 (first three cardiac cycles after apnea begins) and A2 (last three cardiac cycles before breathing resumes). Room air A2 is represented as a black bar and hyperoxia time match is represented as a white bar. Base, baseline; MVEEA, maximal voluntary end expiratory apnea; MAP, mean arterial pressure; HR, heart rate; CVC, cutaneous vascular conductance; BBFV, brachial blood flow velocity. Data are presented as M ± SEM, *denotes significant difference between gases, *P* < 0.05.

**Figure 3.**
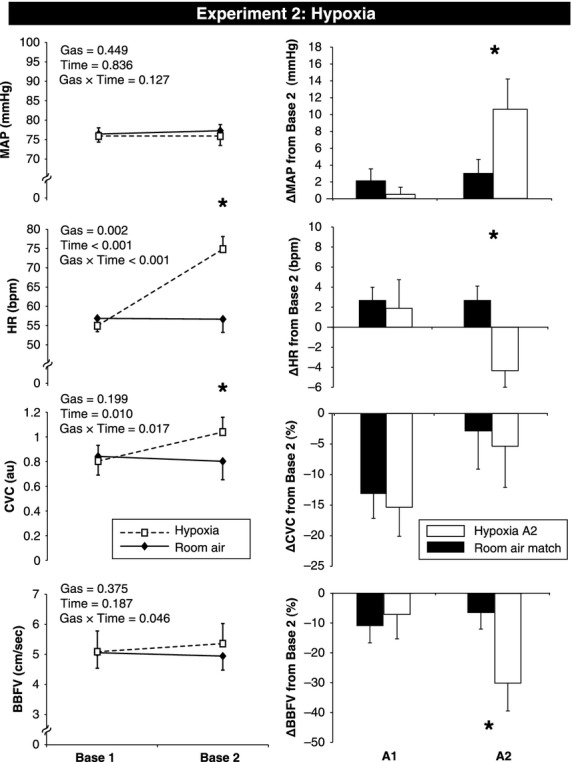
Hemodynamic and vascular changes in response to hypoxic gas and room air apnea in Experiment 2. Absolute changes from Base 1 (room air) to Base 2 (at the end of hypoxia or room air administration) are presented in the left panel. Room air is represented by a solid line, and hypoxia is represented by a dashed line. Changes from Base 2 are presented in the right panel for both A1 (first three cardiac cycles after apnea begins) and A2 (last three cardiac cycles before breathing resumes). Room air time match is represented as a black bar, and hypoxia A2 is represented as a white bar. Base, baseline; MVEEA, maximal voluntary end expiratory apnea; MAP, mean arterial pressure; HR, heart rate; CVC, cutaneous vascular conductance; BBFV, brachial blood flow velocity. Data are presented as M ± SEM, *denotes significant difference between gases, *P* < 0.05.

**Figure 4.**
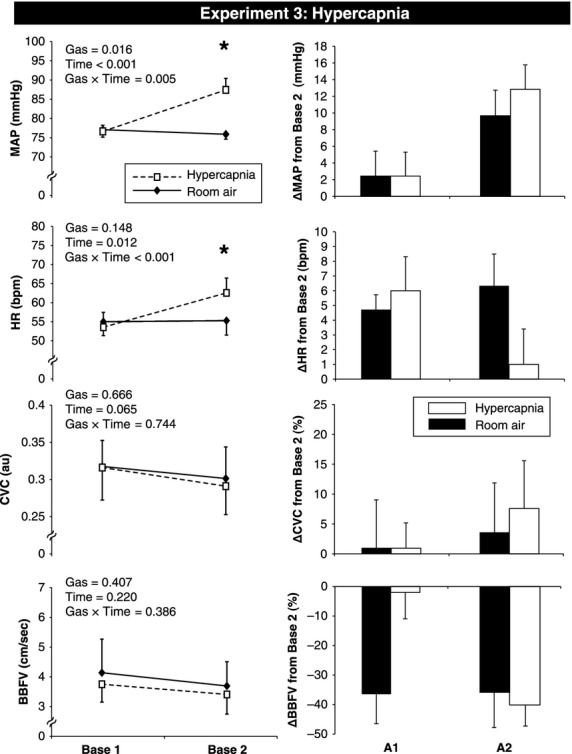
Hemodynamic and vascular changes in response to hypercapnic gas and room air apnea in Experiment 3. Absolute changes from Base 1 (room air) to Base 2 (at the end of hypercapnia or room air administration) are presented in the left panel. Room air is represented by a solid line, and hypercapnia is represented by a dashed line. Changes from Base 2 are presented in the right panel for both A1 (first three cardiac cycles after apnea begins) and A2 (last three cardiac cycles before breathing resumes). Room air is represented as a black bar, and hypercapnia is represented as a white bar. Base, baseline; MVEEA, maximal voluntary end expiratory apnea; MAP, mean arterial pressure; HR, heart rate; CVC, cutaneous vascular conductance; BBFV, brachial blood flow velocity. Data are presented as M ± SEM, *denotes significant difference between gases, *P* < 0.05.

To evaluate the effects of each gas at rest (prior to MVEEA), repeated measures analysis of variance (ANOVA) was used followed by the uncorrected paired samples *t*‐tests. Changes from Base 2 are presented in Figures 2–4. Data are presented as Mean ± SEM and *P* values < 0.05 were considered statistically significant.

## Results

### Experiment 1: Voluntary end expiratory apneas under hyperoxia versus room air

As shown in Figure 2 (left panel), breathing 100% oxygen for 2 min did not have a significant effect on MAP, HR, CVC, or BBFV. With hyperoxia, minute ventilation was unchanged (8.25 ± 0.5 to 7.89 ± 0.6 L), ETCO2 was unchanged (42 ± 1 to 42 ± 1 mmHg), and SaO_2_ increased from 97 ± 1 to 99 ± 1% (*P* < 0.001 vs. Base 1). Expectedly, the breath hold duration was significantly longer following exposure to hyperoxia compared to room air (69 ± 16 vs. 35 ± 8 sec, *P* = 0.027) and the O_2_ saturation nadir was also significantly higher (hyperoxia: 98 ± 0.3 vs. room air: 92 ± 1%, *P* < 0.001). Thus, “time matched control” data are presented for hyperoxia in Figure 2 (right panel) and R1 data could not be statistically compared. Hemodynamic and vascular responses to MVEEA were not different between room air and hyperoxia at A1. However, at A2, ΔMAP (*P* = 0.015), ΔCVC (*P* = 0.033), ΔBBFV (*P* = 0.035), and FVCI (−16 ± 10 vs. −40 ± 12%, *P* = 0.023) were attenuated by hyperoxia. Thus, inhibition of the carotid chemoreceptors with brief 100% oxygen inhalation attenuated both skin vasoconstriction and skeletal muscle vasoconstriction in response to voluntary apnea.

### Experiment 2: Voluntary end expiratory apneas under hypoxia versus room air

Figure 3 (left panel) displays that breathing 10% oxygen for 5 min caused a significant rise in HR and CVC. BBFV also showed a gas × time interaction, indicating an increased response with hypoxia. With hypoxia, minute ventilation increased from 8.68 ± 1.15 to 11.23 ± 2.02 L (*P* < 0.001), ETCO2 decreased from 41 ± 1 to 37 ± 2 mmHg (*P* < 0.001), and SaO_2_ decreased from 97 ± 1 to 80 ± 2% (*P* < 0.001 vs. Base 1). Expectedly, the breath hold duration was significantly shorter following exposure to hypoxia compared to room air (18 ± 2 vs. 35 ± 4 sec, *P* < 0.001) and the O_2_ saturation nadir was also significantly lower (hypoxia: 66 ± 4 vs. room air: 90 ± 1%, *P* < 0.001). Thus, “time matched control” data are presented for room air in Figure 3 (right panel) and R1 data could not be statistically compared. Hemodynamic and vascular responses to MVEEA were not different between room air and hypoxia at A1. At A2, ΔMAP (*P* = 0.047), ΔBBFV (*P* = 0.031), and FVCI (−38 ± 9 vs. −20 ± 5%, *P* = 0.05) were augmented by hypoxia. However, CVC responses to MVEEA were not different (*P* = 0.709). Thus, activation of the carotid chemoreceptors with 5 min of 10% oxygen inhalation raised skin blood flow at rest but the cutaneous vasoconstrictor response to apnea was not enhanced by hypoxia (whereas skeletal muscle vasoconstriction was enhanced by hypoxia).

### Experiment 3: Voluntary end expiratory apneas under hypercapnia versus room air

Figure 4 (left panel) shows that breathing hypercapnia for 5 min caused a significant rise in MAP and HR compared to breathing room air. With hypercapnia, minute ventilation increased from 9.41 ± 1.5 to 26.7 ± 2.7 L (*P* < 0.001), ETCO2 increased from 43 ± 1 to 58 ± 3 mmHg (*P* < 0.001) and SaO_2_ was unchanged (97 ± 1 to 98 ± 1%). The breath hold duration was similar between hypercapnia (38 ± 4 sec) and room air (33 ± 3 sec, *P* = 0.368). Therefore, A2 of each trial was statistically compared (i.e., no “time matched control” data were used). As expected, the O_2_ saturation nadir was significantly different between hypercapnia (98 ± 0.5%) versus room air (90 ± 1%, *P* < 0.001). Hemodynamic and vascular responses to MVEEA were not different between room air and hypercapnia at A1 or A2 (Fig. 4, right panel). At R1, there was no significant difference in MAP (15 ± 3 vs. 15 ± 3 mmHg, *P* = 0.783), BBFV (−49 ± 7 vs. −42 ± 12%, *P* = 0.496), FVCI (−53 ± 7 vs. −48 ± 5%, *P* = 0.614), or CVC (3 ± 10 vs. 13 ± 16%, *P* = 0.692) between hypercapnia and room air, respectively. Thus, activation of the central chemoreceptors with 5 min of hypercapnia inhalation raised HR and BP at rest but had no effect on the forearm vascular responses to voluntary apnea.

## Discussion

The purpose of this experiment was to evaluate forearm vascular responses (skin vs. skeletal muscle) to peripheral and central chemoreflex activation. To avoid the confounding effects of lung inflation, subjects performed MVEEA which itself causes a reduction in SaO_2_ and a rise in end tidal CO_2_ under control (room air) conditions. Since the duration of MVEEA was different between gases (Experiments 1 and 2), we compared peak physiological responses of the shorter trial to the “time matched control” of the longer trial. Consistent with our hypothesis for Experiment 1, peripheral chemoreflex inhibition with hyperoxia attenuated the cutaneous vasoconstrictor response to MVEEA. Contrary to our hypothesis for Experiment 2, peripheral chemoreflex activation with hypoxia did not augment the vasoconstrictor response in skin, but did in muscle. Consistent with our hypothesis for Experiment 3, central chemoreflex activation with hypercapnia did not augment the cutaneous vasoconstrictor response. These results advance our understanding of how chemoreflexes influence vascular regulation in skin and muscle. Specifically, skin blood flow is controlled by the peripheral chemoreceptors but not the central chemoreceptors.

### Experiment 1 Voluntary end expiratory apneas under hyperoxia versus room air

Previous studies have used brief hyperoxia inhalation as a way to deactivate the peripheral chemoreceptors (Izdebska et al. 1996, 1998; Stickland et al. 2008) and examine sympathetic and muscle blood flow responses to physiologic stress. In this study vasoconstriction in the forearm, measured by either BBFV or FVCI, was attenuated by hyperoxia (Fig. 2). This result was expected based on previous literature, and we provide the novel finding that cutaneous vasoconstriction was also attenuated. This difference was not observed at A1 which is to be expected since SaO_2_ has not been reduced from Base 2. To our knowledge, this is the first laboratory experiment to show that the skin vasoconstrictor response to apnea is attenuated following brief hyperoxia inhalation. We purposely administered hyperoxia for only 2 min to avoid baseline changes in forearm blood flow (Fig. 2). Yamazaki et al. (2007) demonstrated that 5 min of hyperoxia caused an ~25% reduction in CVC and previous studies have shown that 5 min of hyperoxia decreases muscle and coronary flow as well (McNulty et al. 2007; Gao et al. 2012; Muller et al. 2013a). Our methodology allowed us to avoid a “basement effect” and demonstrate that the peripheral chemoreflex does, in fact, influence skin blood flow regulation during a voluntary apnea. To summarize Experiment 1, our brief administration of hyperoxia selectively inhibited the carotid chemoreceptors and attenuated vasoconstriction in both muscle and skin in response to MVEEA.

### Experiment 2: Voluntary end expiratory apneas under hypoxia versus room air

Hypoxia has been widely used to study respiratory and cardiovascular physiology in humans (Leuenberger et al. 1995, 2005; Smith et al. 1996). Recent studies from our laboratory have demonstrated that CVC, BBFV, HR, and minute ventilation all increase in response to 5 min of breathing continuous hypoxia (Muller et al. 2013b; Pollock et al. 2014). Moreover, it has been shown that hypoxia augments the sympathetic and forearm vasoconstrictor response (only muscle blood flow) to voluntary apnea (Hardy et al. 1994; Leuenberger et al. 2001). Our data in Figure 3 are consistent with these previous studies, and we also provide novel insight into the cutaneous circulation. Specifically, hypoxia did not cause any significant changes in CVC at A1 or A2 compared to room air. The data from Experiment 2 suggest that hypoxic vasodilation in the skin offsets apnea‐induced cutaneous vasoconstriction.

### Experiment 3: Voluntary end expiratory apneas under hypercapnia versus room air

In Experiment 3, we intentionally gave a hypercapnic gas mixture that would not stimulate peripheral chemoreceptors (due to 30% O_2_ content) in order to selectively study central chemoreceptor effects on forearm blood flow. Narkiewicz et al. showed that central chemoreceptors are more sensitive in obesity (Narkiewicz et al. 1999a) and heart failure (Narkiewicz et al. 1999b), whereas peripheral chemoreceptors are more sensitive in obstructive sleep apnea (Narkiewicz et al. 1999c). Thus, understanding how different chemoreceptor subtypes affect the vasculature may have clinical relevance. With respect to acute blood flow responses, it has been shown that hypercapnia causes vasodilation in the skeletal muscle and cerebral circulations (Kontos et al. 1967; Bishop et al. 1986). However, skin blood flow responses to hypercapnia are less pronounced. Indeed, Wingo et al. (2008) demonstrated that 5 min of hypercapnia had no significant effect on CVC, whereas Simmons et al. (2007) demonstrated a small but significant increase in CVC in response to 5 min of hypercapnia. Our data (Fig. 4, left panel) indicate that 5 min of hypercapnia had no effect on CVC or BBFV at Base 2. Additionally, the hemodynamic and vascular responses to MVEEA were not different between hypercapnia and room air. In fact, we did not observe cutaneous vasoconstriction under room air conditions of Experiment 3 (*n* = 7) which conflicts with the room air data from Experiments 1 and 2 and also our prior publication (Patel et al. 2014). Evaluating individual differences shows that five of the seven young men did experience vasoconstriction but one vasodilated and one did not change. Taken together, this indicates that peripheral and central chemoreceptors have different effects on the cutaneous circulation.

Although skin blood flow may be easier to measure than other vascular beds, it may not be representative of the whole circulatory system. In this study with body temperature rigorously controlled, it appears that skin and muscle blood flow respond similar to MVEEA in room air (i.e., both vasoconstrict to a similar degree). However, the contribution of central and peripheral chemoreceptors to this response is not identical. Thus, it is incorrect to assume that skin and muscle blood flow always respond similar to sympathetic activation.

## Conclusions

In this study, we observed that peripheral chemoreceptor inhibition with brief hyperoxia blunted reflex cutaneous vasoconstriction in response to voluntary apnea (Experiment 1). However, continuous hypoxia (Experiment 2) and continuous hypercapnia (Experiment 3) did not affect the cutaneous vasoconstrictor response to voluntary apnea. These findings expand on previous research and suggest that skin and muscle blood flow are controlled by different reflex pathways. The current data may also encourage further study of how chemoreflex activation influences vascular control in obesity, sleep apnea, and heart failure.

## Acknowledgments

The authors are grateful for the nursing support provided by C. Blaha, J. Mast, T. Nicklas, and A. Caufmann and the engineering support provided by M. Herr. H. Patel, B. Randolph, and A. Ross all contributed to data collection. Gratitude is also extended to A. Muller for preparing the graphics for this study and to L. Sinoway and U. Leuenberger for clinical oversight and funding support. Finally, the authors acknowledge the administrative guidance of K. Gray and J. Stoner.

## Conflict of Interest

There are no conflicts of interest.
